# Imbalanced mitochondrial dynamics in human PD and α-synuclein mouse brains

**DOI:** 10.1016/j.nbd.2025.106976

**Published:** 2025-05-26

**Authors:** Harry J. Brown, Rebecca Z. Fan, Riley Bell, Said S. Salehe, Carlos Martínez Martínez, Yanhao Lai, Kim Tieu

**Affiliations:** a College of Arts and Sciences, Florida International University, Miami, FL, United States of America; b Department of Environmental Health Sciences, Florida International University, Miami, FL, United States of America; c Biomolecular Sciences Institute, Florida International University, Miami, FL, United States of America; d College of Biological Sciences, University of California Davis, Davis, CA, United States of America

**Keywords:** Parkinson’s disease, α-synuclein, Dynamin-related protein 1, Mitochondrial dynamics, Protein aggregation, Neurodegeneration

## Abstract

Emerging studies have shown that dysregulation in mitochondrial dynamics has a major negative impact on mitochondria. Partial genetic and pharmacological inhibition of the mitochondrial fission dynamin-related protein 1 (DRP1) has been demonstrated to be beneficial in models of neurodegenerative disorders, including Parkinson’s disease (PD). However, the expression of DRP1 and other mitochondrial fission/fusion mediators have not been investigated in the brains of Parkinson’s patients. This information is critical to strengthening mitochondrial dynamics as a potential therapeutic target for PD. We report in this study that significant increases in the levels of both *DNM1L,* which encodes DRP1, as well as the DRP1 protein were detected in Parkinson’s patients. Immunostaining revealed increased DRP1 expression in dopamine (DA) neurons, astrocytes, and microglia. In addition to DRP1, the levels of other fission and fusion genes/proteins were also altered. To complement these human studies and given the significant role of α-synuclein in PD pathogenesis, we performed time-course studies using transgenic mice overexpressing human wild-type *SNCA*. As early as six months old, we detected an upregulation of DRP1 in the nigral DA neurons of the *SNCA* mice as compared to their wild-type littermates. Furthermore, these mutant animals exhibited more DRP1 phosphorylation at serine 616, which promotes its translocation to mitochondria to induce fragmentation. Together, this study shows an upregulation of DRP1 and alterations in other fission/fusion proteins in both human and mouse PD brains, leading to a pro-fission phenotype, providing additional evidence that blocking mitochondrial fission or promoting fusion is a potential therapeutic strategy for PD.

## 1. Introduction

Parkinson’s disease (PD) is the second most prevalent and the fastest growing neurodegenerative disease (Dorsey et al., 2018). This debilitating neurological disorder is characterized pathologically by the presence of toxic α-synuclein (α-syn) and loss of dopamine (DA) neurons in the substantia nigra pars compacta (SNpc). Since its first discovery almost three decades ago in familial PD (Polymeropoulos et al., 1997), α-syn has increasingly received attention due to its prominent role, not only in familial but also in sporadic PD. The significance of this protein is further demonstrated when it was recently reported to be a reliable biomarker for PD (Siderowf et al., 2023). Aggregation of this small protein has long been found in the Lewy bodies (LBs) (Spillantini et al., 1997), which are intracellular inclusions considered to be the histological hallmark of PD. Although it is still a topic of debate whether LBs are neurotoxic or neuroprotective by sequestering the toxic oligomeric α-syn (Chartier and Duyckaerts, 2018; Parkkinen et al., 2011), a recent study reported that widespread non-inclusion pathology of toxic α-syn oligomers occurs prior to the formation of LBs (Jensen et al., 2024). The neurotoxic effects of α-syn have also been demonstrated experimentally to spread from one cell to another in a prion-like fashion (Peng et al., 2020; Pinnell et al., 2021). Overall, abnormal conformation of α-syn is neurotoxic in PD.

Several pathogenic mechanisms associated with α-syn have been proposed, ranging from mitochondrial dysfunction, impaired protein degradation pathways (autophagy and ubiquitin proteasomal system), oxidative stress, synaptic dysfunction, and neuroinflammation. These mechanisms are not mutually exclusive and impairing one pathway such as mitochondrial function can result in activation of other pathogenic mechanisms (Helley et al., 2017). The negative impact of α-syn has been demonstrated experimentally in both *in vitro* and *in vivo* models (Bido et al., 2017; Devi et al., 2008; Di-Maio et al., 2016; Fan et al., 2019; Subramaniam et al., 2014) by impairing mitochondrial respiration. These studies primarily focus on the impact of α-syn on the mitochondrial electron transport chain. However, some *in vivo* studies have also reported that α-syn overexpression induces mitochondrial fragmentation (Bido et al., 2017; Chen et al., 2015), suggesting imbalance of mitochondrial dynamics leading to a pro-fission phenotype.

Mitochondrial dynamics refers to the continuous process of fission (division), fusion (joining) and movement of mitochondria. It is now recognized that a balance in mitochondrial fusion and fission is critical to neuronal function and viability. Fission and fusion are regulated by specific proteins. The outer mitochondrial mitofusin proteins (MFN1 and MFN2) and the inner mitochondrial protein optic atrophy 1 (OPA-1) are responsible for mitochondrial fusion. Mitochondrial fission is controlled by a separate set of proteins: Mitochondrial Fission Factor (MFF), Fission-1 (FIS1), as well as Mitochondrial Dynamics Proteins of 49 and 51 kDa (MID49 and MID51, respectively) are anchored to the outer mitochondrial membrane (OMM) where they recruit cytosolic Dynamin-related protein-1 (DRP1), which then oligomerizes and forms a ring-like structure around the mitochondria to constrict and divide them (Chan, 2020; Knott and Bossy-Wetzel, 2008; Oliver and Reddy, 2019). Although the role of DRP1 in mitochondrial division is critical to normal cellular function, excessive mitochondrial fission is detrimental to cells. Accumulating evidence indicates that partial inhibition of DRP1 function is protective in experimental models of neurodegenerative diseases (Archer, 2013; Oliver and Reddy, 2019). However, the expression of DRP1 has not been investigated in the brain samples of Parkinson’s patients. This information is critical if this mitochondrial fission is to be considered as a potential therapeutic target for PD. In the present study we provide data showing the expression of DRP1 and other mitochondrial fission and fusion proteins/genes in the substantia nigra (SN) post-mortem samples of Parkinson’s patients. To complement these findings, we performed time-course studies in transgenic mice overexpressing human wild-type α-syn under the *Thy1* promoter (Choi et al., 2020; Polinski et al., 2022). Together, these results show an upregulation of DRP1 and alterations in other fission/fusion proteins leading to a pro-fission phenotype.

## 2. Materials and methods

### 2.1. Animals

All animals used in this study were approved by the Institutional Animal Care and Use Committee at Florida International University. Mice expressing human *SNCA* under the *Thy-1* promoter [C57BL/6 N-Tg (Thy1-SNCA)15Mjff/J, strain #017682] are commercially available from the Jackson Laboratory. Hemizygotes were crossed with C57BL/6 J (Jackson Laboratory, strain #000664) mice to establish and maintain the colony. Heterozygous DRP1-knockout mice were generated and maintained as previously described (Fan et al., 2024). To generate double mutant mice with heterozygous overexpression of α-syn and heterozygous DRP1-knockout mice, we crossed *SNCA*^+/−^ mice with *Dnm1l*^+/−^ mice.

### 2.2. Human tissue

All postmortem samples used were obtained from the NIH NeuroBioBank. Inclusion criteria for individuals with PD are clinical diagnosis and neuropathology (Lewy bodies and dopaminergic neurodegeneration in the substantia nigra). Co-morbidities are excluded for both Parkinson’s patients and control subjects. For more information, please refer to Supplementary Table 1.

### 2.3. Simple western capillary immunoblotting

Capillary immunoblotting was performed with Jess^™^ Simple Western system (ProteinSimple, Bio-Techne) using the 12–230 kDa Fluorescence Separation Module (cat# SM-FL004 ProteinSimple, Bio-Techne), as previously described (Fan et al., 2024). Briefly, cytosolic fractions were isolated from frozen pulverized human tissue by homogenization with cytoplasmic extraction buffer (10 mM HEPES, 60 mM KCl, 1 mM EDTA, 0.075 % IPEGAL, 1 mM PMSF and 1 mM DTT, pH 7.6). Incubation at 4 °C, rotating for 10 mins prior to centrifugation at 200 ×*g* to collect cytosolic supernatant. Mouse micro dissected ventral midbrains were homogenized in Triton extraction buffer as previously described (Stojkovska and Mazzulli, 2021) ([1 %] Triton X-100, [150 mM] NaCl, [20 mM] HEPES pH 7.4, [1 mM] EDTA, [1.5 mM] MgCl2, [10 %] glycerol, [50 mM] NaF, [2 mM] Na3VO4, [0.5 mM] PMSF, and [1×] protease inhibitor cocktail, followed by incubating in an ice water slurry for 30 min with gentle mixing on a shaker. Lipid bilayers were disrupted by three freeze-thaw cycles of submerging samples in −80 °C ethanol bath for 2 mins followed by 37 °C water bath for 1 min. Lysates were then ultracentrifuged at 100,000 ×g for 30 mins at 4 °C and soluble supernatant was collected.

Micro BCA protein assay (23235, Thermo Fisher Scientific) was used to determine sample protein concentration, 1.5 μg protein of human samples was loaded per capillary and 3 μg of mouse samples. Primary antibodies used: DRP1 (Cell signaling, 8570S, 1:200), MID51 (Proteintech, 67808–1-Ig, 1:1000), MFN2 (Sigma, M6319, 1:200), OPA-1 (BD transduction laboratories, 612606, 1:200), GAPDH (Cell signalling, 2118, 1:200). Sample run parameters were: Separation time 25 mins. Primary incubation 60 mins. Secondary incubation 30 mins. Replex (blot stripping) time 30 mins. Due to the potential concern of overlapping signal between MID51 and GAPDH based on their similar molecular weight, we used mouse MID51 and rabbit GAPDH primary antibodies. Additionally, using Jess capillary blotting, the target with the weakest signal (MID51) was detected in probe 1 and the strongest signal marker (GAPDH) was detected in probe 2. This is to ensure that when the capillary is stripped no residual signal from probe 1 is detectable. As confirmed by the data plots from capillary immunoblotting shown in Supplementary Fig. S1, the emission peaks from each chemiluminescent marker do not overlap and there is no secondary emission peak at the molecular weight of MID51 in probe 2.

### 2.4. RNA extraction and quantitative RT-PCR

Total RNA was extracted from human or mouse ventral midbrain using TRIzol (Thermo Fisher Scientific) per manufacturer’s instructions. RNA (500 ng) was converted to complementary DNA (cDNA) using iScript Reverse Transcription Supermix (Bio-Rad). A QuantStudio 6 detection system (Thermo Fisher Scientific) was used for qPCR reaction using TaqMan assays and TaqMan Fast Advanced Master Mix (Thermo Fisher Scientific). Reaction conditions were 50 °C for 2 mins, 95 °C for 2 mins and 40 cycles of 95 °C for 1 s and 60 °C for 20s. The 2^−ΔΔCT^ method was used for quantification. TaqMan assays used for human samples were *GAPDH* (HS02786624_g1), *DNM1L* (HS01552605_m1), *MFF* (HS00697394_g1), *MIEF2* (HS00541009_g1), *MIEF1* (HS01007730_g1), *OPA-1*: (HS01047013_m1), and *MFN2* (HS00208382_m1); for mouse studies were *Gapdh (*Mm99999914_g1), *Dnm1l* (Mm01342903_m1), *Mff* (Mm01273401_m1), *Mief1* (Mm00724569), *Mief2* (Mm01234249_g1), *Opa-1* (Mm01349707_g1), and *Mfn2* (Mm00500120_m1).

### 2.5. Immunostaining

#### 2.5.1. Human postmortem brain sections

Antigen retrieval: pre-mounted SN sections 10 μm thick were deparaffinized overnight at 60 °C. Rehydration was performed sequentially using: [100 %] xylene, [100 %] ethanol, [95 %] ethanol, [70 %] ethanol, [50 %] ethanol and distilled water. Antigen retrieval was performed using Antigen Unmasking Solution (Vector, H-3300) and boiled in a steamer for 20 mins before cooling at room temperature (RT) for 30 mins.

Immunofluorescence: deparaffinized sections were washed in [1×] PBS, followed by permeabilization in [10 %] Triton-X for 5 mins. Sections were washed in [1×] PBS before incubation in [1 %] Sudan black solution in [70 %] ethanol for 2 h at RT. Subsequently sections were incubated overnight at 4 °C with primary antibody solution ([4 %] normal goat serum (NGS), [0.5 %] Triton-X, [0.05 %] Tween-20, and [1×] PBS) contained within a humidity chamber. Sections were washed in [1×] PBS and incubated in secondary antibody solution for 1 h at RT ([4 %] NGS in [1×] PBS). Sections were washed in [1×] PBS and mounted using prolong gold antifade reagent with DAPI (Thermo Fisher Scientific, #P36941).

Immunohistochemistry: Brain sections were deparaffinized as described above and washed in [0.1 M] TBS. Sections were then permeabilized and endogenous peroxidases was quenched with [0.3 %] hydrogen peroxide in methanol for 10 mins with shaking. Sections were washed in [0.1 M] TBS before blocking for 1 h at RT using [5 %] NGS in [0.1 M] TBS. Tissue sections were then incubated in corresponding primary antibodies overnight at 4 °C in [0.1 M] TBS containing [2 %] NGS. Hereafter tissue sections were washed in [0.1 M] TBS and incubated for 1 h at RT in corresponding biotinylated secondary antibody (1:200) in [0.1 M] TBS with [2 %] NGS. Sections were washed in 0.1 M TBS before incubation for 1 h at RT in ABC solution according to manufacturer’s guidelines (Vector, PK6100). ABC solutions were made 30mins prior to usage. Sectionswere washed in [0.1 M] TBS. Color generation was performed using DAB substrate kit, with nikel enhancement according to manufacturer’s guidelines (Vector, SK-4100). Sections were dehydrated in [95 %] ethanol, [100 %] ethanol and xylene before being sealed using DPX Mountant (Sigma, 06522). Primary antibodies used were DRP1 (BD biosciences, 6111133, 1:500), IBA1 (Wako, 019–19,741, 1:1000), GFAP (Invitrogen, PA1–10004, 1:20,000).

#### 2.5.2. Mouse brain sections

Mice were transcardially perfused, stored and sectioned as previously described (Fan et al., 2024). Ventral midbrain sections (30 μm) were washed in [0.1 M] TBS. Non-specific binding sites were blocked for 1.5 h at RT using [4 %] NGS and [0.5 %] Triton-X in [0.1 M] TBS. Primary antibodies were diluted in [2 %] NGS, [0.3 %] Triton-X in [0.1 M] TBS and incubated overnight at 4 °C whilst shaking. Sections were washed in [0.1 M] TBS and incubated in secondary antibodies for 1 h at RT whilst shaking. Primary antibodies: DRP1(BD biosciences, 6111133, 1:500), Iba1 (Wako, 019–19,741, 1:1000), GFAP (Invitrogen, PA1–10004, 1:5000), TH (Abcam, ab76442, 1:2000), and TOMM20 (Abcam, ab186735, 1:1000). Secondary antibodies: Alexa Fluro 488 goat anti-mouse IgG (H + L) (Invitrogen, A11029, 1:1000), Alexa Fluro 647 goat anti-chicken IgY (Invitrogen, 1016315–1, 1:1000), Alexa Fluro 568 goat anti-rabbit IgG (H + L) (Invitrogen, A11011, 1:1000).

### 2.6. Mitochondrial network analysis

Mitochondrial morphology was quantified using Mitochondrial Network Analysis (MiNA, a macro toolset of Fiji utilizing existing ImageJ plug-ins) as previously described [38]. Briefly, mitochondria were visualized by immunofluorescence using TOMM20 (Abcam, ab186735). Images were captured and z-stacked using Olympus Fluoview 1200 confocal. Prior to MiNA analysis, 8-bit gray scale images were binarized and skeletonized using the following steps: Process, filters, unsharp mask; Process, enhance local contrast (CLAHE), and median filtering using Fiji. Process, filters, median; Process, binary, make binary; Process, binary, skeletonize; Analyze, skeleton, analyze 2D/3D skeleton. Hereafter the StuartLab plugin was used to analyze the mitochondrial network using the following parameters. Threshold method: otsu. Maximum threshold: 50. Minimum threshold: 10. Line width: 1. Line length: 5. (Valente et al., 2017). After generating morphological skeletons (2D), the “tagged skeletons” were analyzed using MiNA, which divides objects (mitochondria) into two distinct types: individuals (puncta and rods but no branches) and networks (with connected branches). TH staining channel was used to outline cell soma used for mitochondria analysis.

#### 2.6.1. Imaris 3D rendering

Imaris (Oxford instruments) was used to determine DRP1 and mitochondrial colocalization. Imported confocal images had surfaces created and masked to visualize individual mitochondrial networks. The surfaces’ function was used to create a 3D render of tyrosine hydroxylase (TH)-positive neurons. DRP1 staining inside the 3D render of TH-positive neurons had the spots’ function applied to render DRP1 puncta in 3D. TOMM20 staining localized inside the 3D render of TH-positive neurons had the surfaces’ function applied to 3D render mitochondria. Individual mitochondria were separated using the split touching object’s function and labelled using individual object IDs. Proximity filtering of DRP1 spots touching mitochondrial renders yielded two DRP1 sub-populations: touching mitochondria or not touching mitochondria. Half of the diameter of DRP1 puncta was used as a threshold to determine if DRP1 was touching or not touching mitochondria.

### 2.7. Statistical analysis

All data points are expressed as mean ± SEM and the mean differences between groups were compared using an unpaired *t*-test or the Two-Way Analysis of Variance (Two-Way ANOVA) as indicated in each figure. Data from animal studies were analyzed using either an unpaired t-test for two-group comparison or a Two-Way ANOVA for mean difference comparison between two variables followed by Tukey’s *post hoc* testing. For human data, an unpaired t-test was used to compare the mean difference between Parkinson’s patients and control samples for different markers. To account for potential confounding effects of age and sex, regression analysis was implemented with age and sex as covariates using the *lm()* function of the stats package (for linear models) in the RStudio programming environment (R 4.4.2, RStudio 12.0 Build 467). Potential outlier data points were identified using a combination of Cook’s D, Standardized Residuals, and leverage from the regression results. Identified outlier data points were removed from the subsequent analysis.

## 3. Results

### 3.1. DRP1 is upregulated in the substantia nigra of Parkinson’s patients

**Fig. 1.**
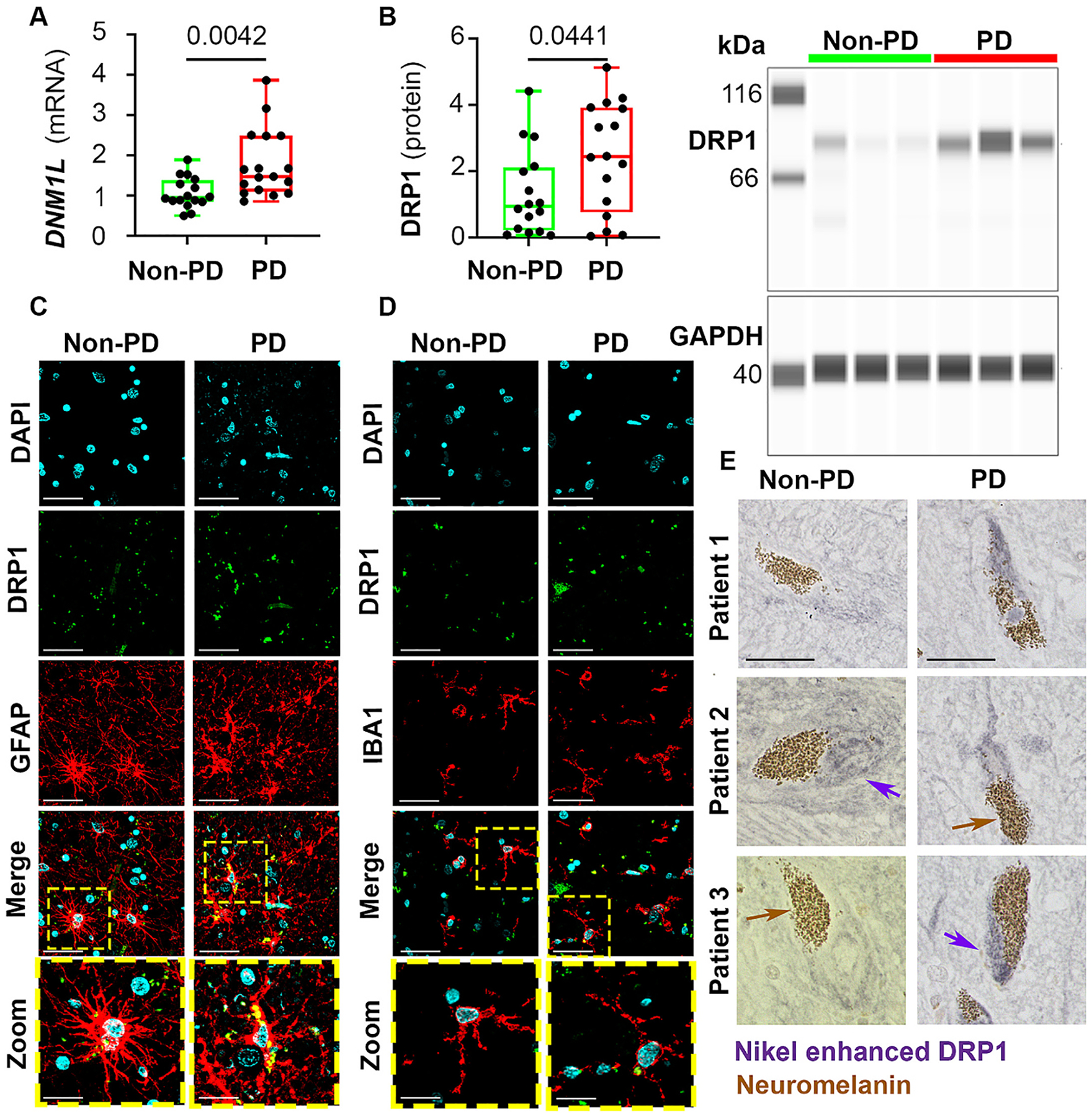
Increased DRP1 levels in human Parkinson’s brains. **(A)** Post-mortem substantia nigra of PD and non-PD subjects were analyzed for the mRNA levels of *DNM1L* using qPCR. **(B)** DRP1 protein levels were assessed in these samples using capillary immunoblotting, which shows three representative non-PD and PD patients of the 16–17 subjects of each group evaluated and quantified. Data were normalized to *GAPDH* mRNA and GAPDH protein, respectively. **(C)** Immunofluorescence imaging of DRP1 levels in astrocytes and **(D)** microglia. **(E)** To avoid autofluorescence from lipofuscin in DA neurons, immunohistochemistry using diaminobenzidine with nickel enhancement was performed to visualize DRP1 (purple arrows) in neuromelanin- (brown arrows) containing DA neurons. Three representative non-PD and PD patients are illustrated. Scale bar = 20 μm for **C-E**. Data represent mean ± SEM, unpaired *t*-test, *n* = 16–17. A, *F*_15,16_ = 4.904. B, *F*_15,15_ = 1.640.

Imbalanced mitochondrial dynamics has been reported in experimental models of PD (Archer, 2013; Knott et al., 2008; Oliver and Reddy, 2019). However, a pro-fission or fusion phenotype in human Parkinson’s patients brain samples is yet to be established. To address this gap, we obtained thirty-three (17 with PD and 16 without PD) postmortem human SNpc samples from the NIH NeuroBioBank. Please refer to Supplementary Table 1 for more information about these human subjects. Given that partial DRP1 inhibition has been reported to be protective in experimental models of PD (Bido et al., 2017; Cui et al., 2010; Fan et al., 2019; Filichia et al., 2016; Lutz et al., 2009; Rappold et al., 2014; Su and Qi, 2013), we first addressed whether DRP1 was upregulated within the SNpc of postmortem samples of human Parkinson’s patients and whether there were any sex differences. The expression of DRP1 was assessed at both transcriptional (mRNA) and translational (protein) levels. As shown in Fig. 1A and B, significant increases in levels of both the gene that encodes DRP1, *Dynamin-1 like protein* (*DNM1L),* and DRP1 protein were seen in Parkinson’s patients compared with controls. No age and sex differences were detectable between control or Parkinson’s subjects (Supplementary Table 2) and therefore, data were pooled for analysis in Fig. 1A and B. To determine if increases in DRP1 were cell-type specific, we performed immunostaining for astrocytes, microglia and DA neurons (Fig. 1C–1E). DRP1 was expressed in all three cell types and consistent with the increase in gene and protein levels as seen in Fig. 1A and B, Parkinson’s patients had higher immunoreactivity of DRP1 in these cells. Together, these data indicate that DRP1 is significantly increased in Parkinson’s patients at both transcriptional and translational levels and is not apparent to be cell-type specific.

### 3.2. Alterations of other mitochondrial fission and fusion genes/proteins in the nigra of Parkinson’s patients

**Fig. 2.**
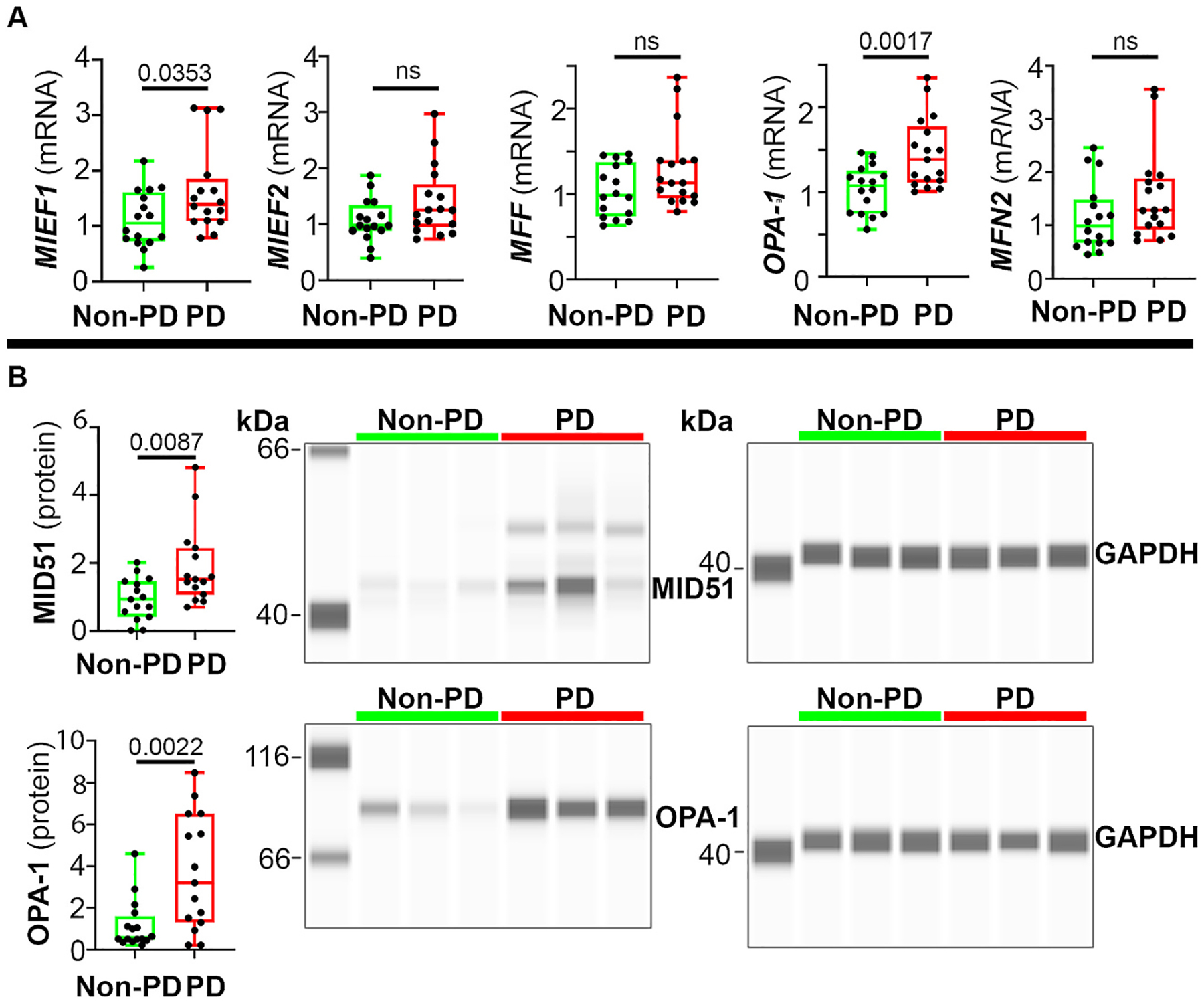
Levels of other mitochondrial dynamics genes and proteins in human brains. **(A)** Post-mortem SN specimens of PD and non-PD subjects were analyzed for gene expression of *MIEF1*, *MIEF2*, *MFF, OPA-1*, and *MFN2* using qPCR. **(B)** MID51 and OPA-1 protein levels were assessed using capillary immunoblotting, which shows three representative non-PD and PD patients. Data represent mean ± SEM, normalized to *GAPDH* or GAPDH. Unpaired t-test, *n* = 15–16. A, *MIEF1 F*_15,15_ = 2.334. *MIEF2 F*_15,16_ = 2.675. *MFF F*_15,16_ = 2.277. *OPA-1 F*_15,16_ = 2.253. *MFN2 F*_15,16_ = 1.731. B, MID51 *F*_14,14_ = 3.577. OPA-1 *F*_14,15_ = 5.470.

To have a more complete profile of whether or how other mitochondrial dynamics related genes and proteins were altered in Parkinson’s patients, we analyzed the expression of other mitochondrial fission and fusion mediators. Transcriptionally, *Mitochondrial Elongation Factor-1* (*MIEF1,* which encodes MID51) and *OPA-1* were significantly upregulated in Parkinsons’s patients (Fig. 2A) as compared to non-PD control subjects. *Mitochondrial Elongation Factor-2* (*MIEF2*), *Mitochondrial Fission Factor* (*MFF)* and *Mitofusin 2* (*MFN2*) levels were not significantly changed in Parkinson’s patients (Fig. 2A). Immunoblotting further confirmed the increases in Mitochondrial Dynamics Protein of 51 kDa (MID51) and OPA-1 protein levels in Parkinson’s patients (Fig. 2B). Overall, these data identify that both pro-fission and pro-fusion genes are differentially expressed in Parkinson’s patients. Such alterations in both fission and fusion factors likely result from a compensatory mechanism.

### 3.3. DRP1 is transiently upregulated in the nigral DA neurons in a PD mouse model

**Fig. 3.**
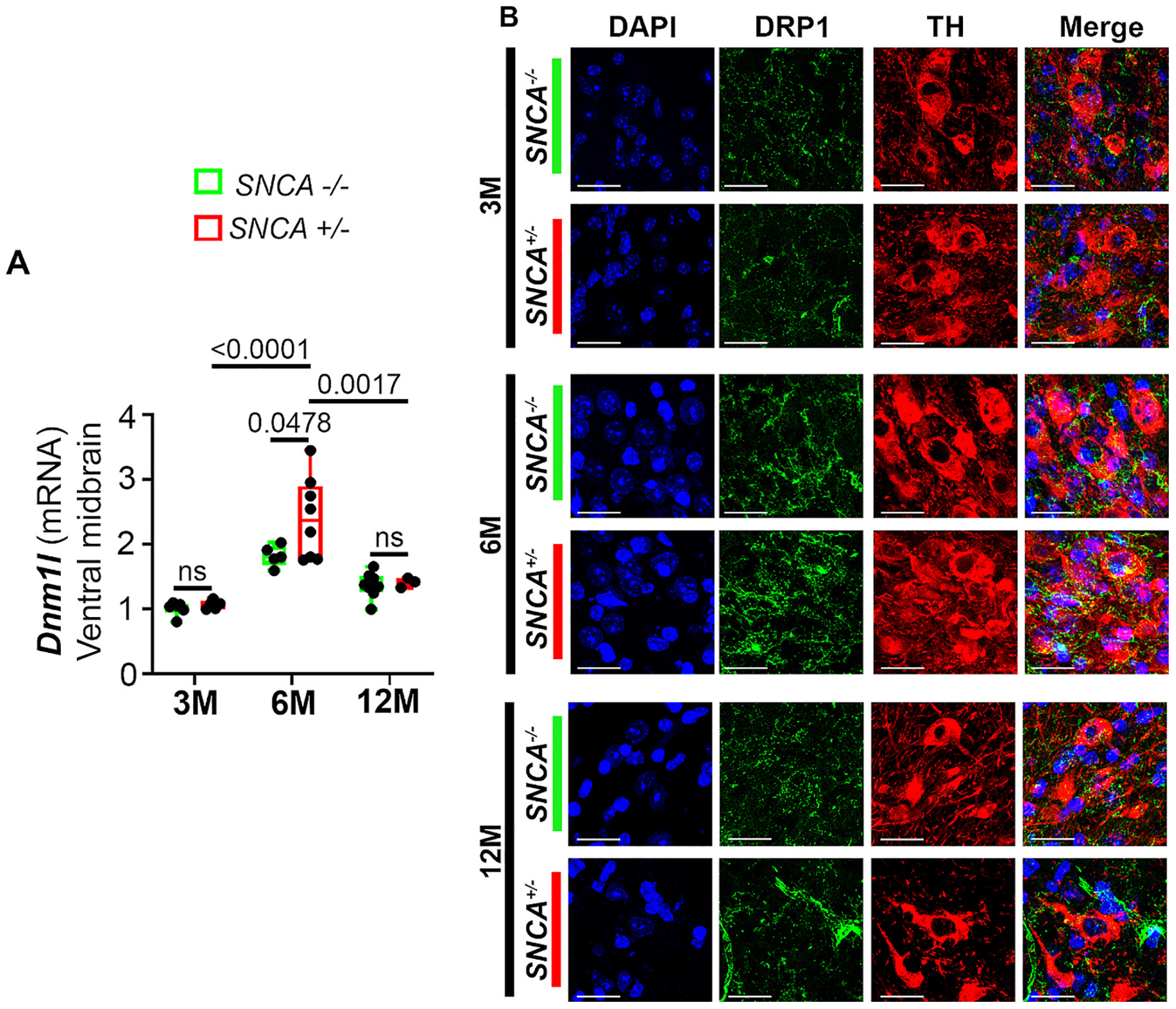
Longitudinal assessment of DRP1 levels in *SNCA* mice. **(A)** Ventral midbrains of heterozygous transgenic mice overexpressing human α-syn (*SNCA*^+/−^) and their WT littermates (*SNCA*^−/−^) at ages 3-, 6-, and 12-months were analyzed for mRNA levels of *Dnm1l* (normalized to *Gapdh*) using qPCR. **(B)** Immunofluorescence imaging of Drp1 colocalizing with TH-positive neurons of the SNpc. Scale bar = 10 μm. Data represent mean ± SEM. Age-dependent and genotype-dependent changes were analyzed using Two-Way ANOVA followed by Tukey’s *post hoc* tests. *n* = 3–8. Two-way ANOVA, age: *F*_2,29_ = 31.42. Genotype *F*_1,29_ = 3.671.

Although translational, there are inherent limitations of using human post-mortem samples. Therefore, we turned to a transgenic mouse model of PD to further investigate the role of DRP1 in the presence of α-syn, a prominent protein involved in PD pathogenesis. With the C57BL/6 N-Tg(Thy1-*SNCA*)15Mjff/J mice, which overexpress human *SNCA* under the *Thy1* promoter (*SNCA*^+/−^) (Choi et al., 2020; Polinski et al., 2022), we performed time-course studies at ages 3-, 6-, and 12-months. At 6 months old, *Dnm1l* gene expression in the *SNCA* mice was upregulated in the ventral midbrain when compared to age-matched WT littermate controls (Fig. 3A). To further investigate whether these changes also occurred in the nigral DA neurons, we performed immunofluorescence staining and detected higher immunoreactivity of DRP1 in TH-positive neurons in 6-month-old *SNCA* mice (Fig. 3B).

To corroborate changes in human Parkinson’s patients with the *SNCA* mouse model, we performed immunoblotting in 3-, 6-, and 12-month-old *SNCA* mice for other mitochondrial fission and fusion markers (MID51, MFN2, and OPA-1) as in the human samples (Supplementary Fig. S2). No significant genotypic changes were detectable between WT and *SNCA* mice in these markers at any age. We did, however, observe age-dependent changes in MID51 and MFN2, both of them peaked in 6-month-old mice before decreasing in 12-month-old mice. OPA-1 levels significantly decreased with age. These findings highlight that overexpression of α-syn did not significantly alter the levels of MID51, MFN2, or OPA-1. Whereas age did significantly alter the levels of these proteins.

In combination, from this *in vivo* time-course study, we discovered that of all the fission and fusion markers that we assessed, DRP1 was most affected by genotypes at 6 months old. The observations of normal levels of DRP1 at 12-months-old suggest that mice have a better ability to combat this increase as compared to Parkinson’s patients. However, this transient increase may be sufficient to induce a negative impact on mitochondria.

### 3.4. SNCA mice exhibit age-dependent mitochondrial fragmentation

**Fig. 4.**
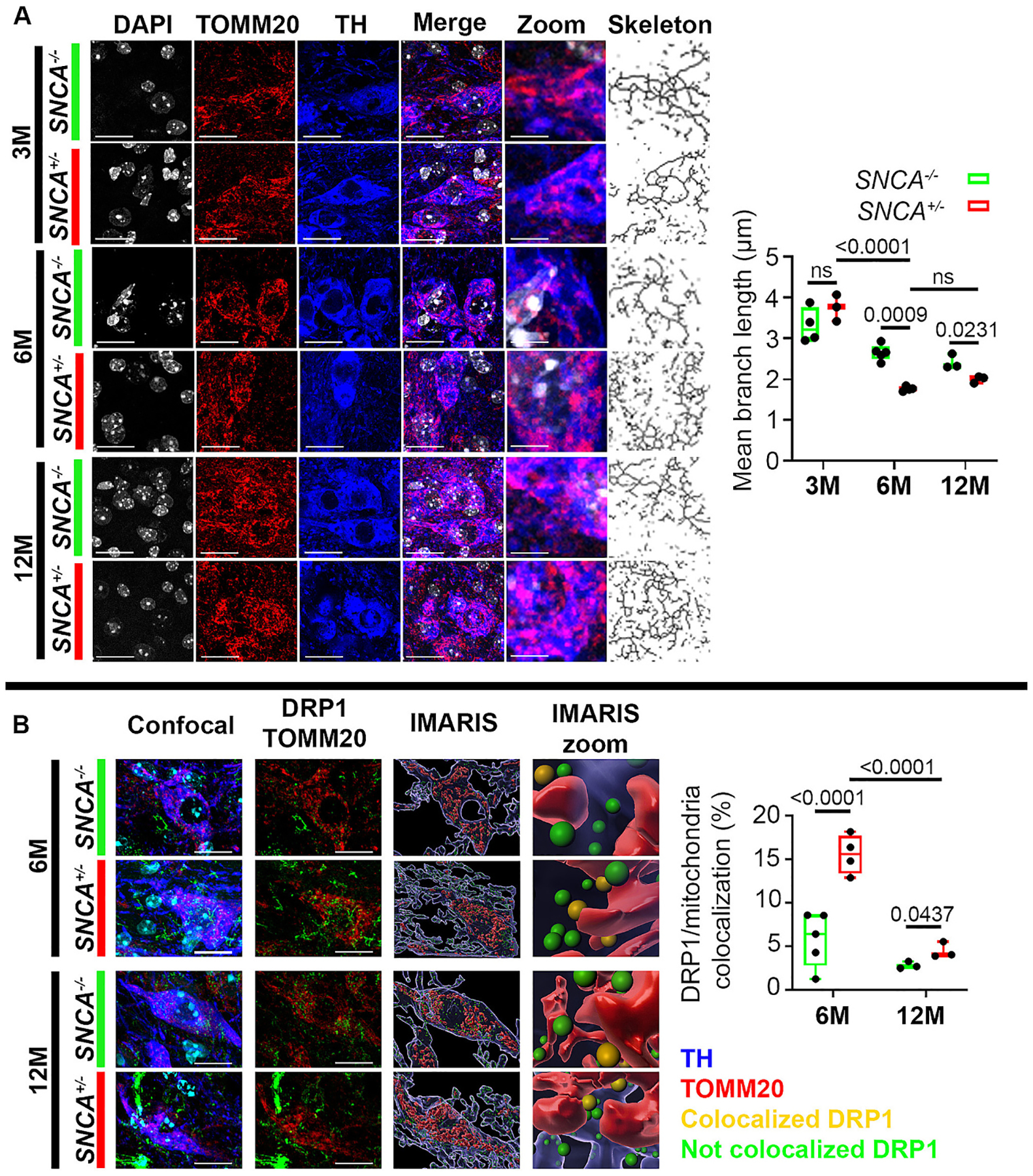
Age-dependent mitochondrial fragmentation in *SNCA* mice. **(A)** Immunofluorescence of TOMM20 to identify mitochondrial morphology in TH-positive neurons of the SNpc in 3-, 6-, and 12-month-old *SNCA* mice and then skeletonized for subsequent analysis of mitochondrial network, scale bar = 10 μm and 2.5 μm. **(B)** Imaris 3D rendering of DRP1 mitochondrial colocalization in 6- and 12-month-old *SNCA* mice. Individual immunofluorescent channels from confocal images were outlined to isolate TH-positive neurons. Zoomed images of IMARIS display IMARIS 3D renders of DRP1 colocalization on mitochondria, Scale bar = 10 μm. Data represent mean ± SEM. Age dependent and genotype dependent changes were analyzed using Two-Way ANOVA followed by Tukey’s *post hoc* tests. Genotypic changes independent of age were analyzed using unpaired t-test, n = 3–5. Two-way ANOVA, age: *F*_2,16_ = 66.07. Genotype: *F*_1,16_ = 6.945. Unpaired t-test, *F*_2,2_ = 4.766.

To further investigate whether increased DRP1 in the 6-month-old transgenic *SNCA* mice would result in a pro-fission phenotype, we colabelled TH-positive neurons in the SNpc of these mice with translocase of outer mitochondrial membrane 20 (TOMM20) to visualize mitochondrial morphology. As shown in Fig. 4A, although mitochondrial fragmentation was observed in both genotypes in an age-dependent manner, *SNCA* mice exhibited more severe fragmentation, as evidenced by a decrease in branch length compared to their WT age-matched littermates (Fig. 4A). To confirm that these changes in mitochondrial morphology were in part attributed to DRP1, we replicated this experiment using 6-month-old double mutant mice with heterozygous overexpression of α-syn and heterozygous DRP1-knockout (Supplementary Fig. S3). As compared to littermates with only α-syn overexpression, double mutant mice exhibited longer mitochondrial branch length, indicative of a protection against the effects of α-syn. To obtain additional evidence that links DRP1 to mitochondrial fragmentation, we performed DRP1 colocalization analysis with mitochondria in TH-positive neurons of the SNpc in 6- and 12-month-old *SNCA* and WT littermates using the 3D rendering software Imaris (Supplementary Fig. S4). This technique allowed us to clearly segregate DRP1 in contact with mitochondria from the mitochondria absent DRP1 population. An increase in DRP1 translocation to mitochondria was detected in 6-month-old *SNCA* mice when compared to age-matched WT controls. Although statistically significant, the extent of DRP1 translocation in the 12-month-old *SNCA* mice was less (Fig. 4b).

Overall, these data demonstrate that there is a significant increase in the expression of DRP1 and its translocation to mitochondria, resulting in severe fragmentation in the nigral TH-positive neurons of the *SNCA* mice, starting at 6-month-old. Additionally, the effects of aging on mitochondrial morphology were further exacerbated in these transgenic mice.

### 3.5. Post-translational modification of DRP1 is increased in SNCA mice

**Fig. 5.**
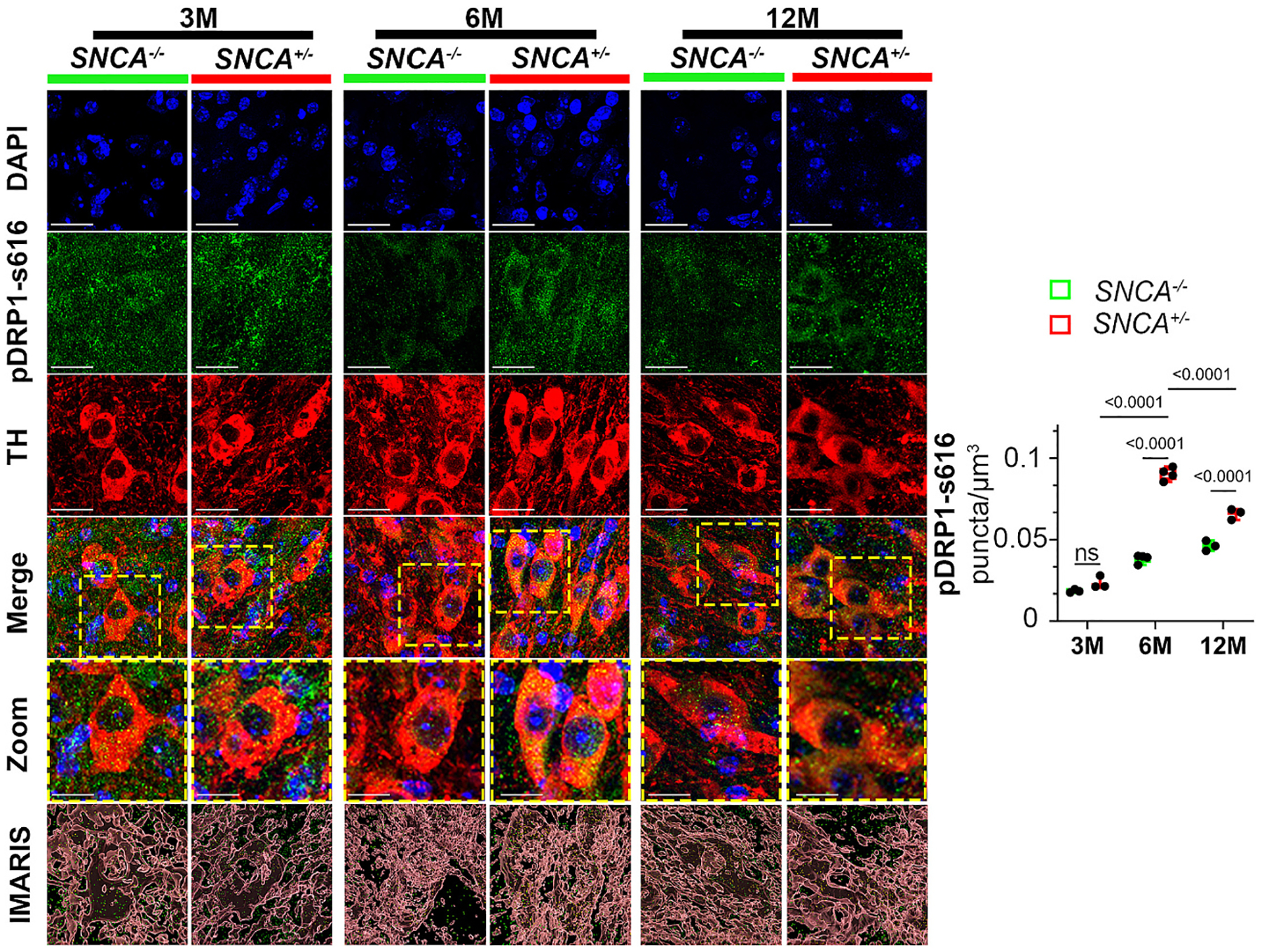
pDRP1-s616 is enriched in TH positive neurons of 6- and 12-month-old *SNCA* mice. Immunofluorescence of pDRP1-s616 in TH-positive neurons of the SNpc. pDRP1-s616 puncta inside TH positive structures were quantified using Imaris 3D rendering. Scale bar = 10 μm. Zoom scale bar = 5 μm. Data represent mean ± SEM. Age dependent and genotype dependent changes were analyzed using Two-Way ANOVA followed by Tukey’s *post hoc* tests. Genotypic changes independent of age were analyzed using unpaired t-test, n = 3–5. Two-way ANOVA, age: *F*_2,14_ = 20.29. Genotype: *F*_1,14_ = 21.22. Unpaired t-test, *F*_2,2_ = 57.53.

One well-established post-translational modification that promotes DRP1 translocation to mitochondria to induce fragmentation is its phosphorylation at the serine 616 residue (pDRP1-s616) (Chan, 2020; Oettinghaus et al., 2012). To investigate whether pDRP1-s616 played a role in mitochondrial fragmentation in the *SNCA* mice, we performed immunofluorescence for pDRP1-s616 in the nigral TH-positive neurons, followed by quantification using Imaris 3D rendering (Supplementary Fig. S5 and Fig. 5). No differences in pDRP1-s616 were detectable between 3-month-old *SNCA* and WT mice. However, pDRP1-s616 was significantly increased in TH-positive neurons of 6-month-old *SNCA* mice, when compared to WT age-matched controls. Although not as extensive, increased pDRP1-s616 was also observed in 12-month-old *SNCA* mice. In aggregate, these results indicate that *SNCA* mice increase post-translational phosphorylation of DRP1 at a site that promotes its translocation to mitochondria to induce mitochondrial fragmentation.

## 4. Discussion

Balanced mitochondrial fission and fusion is essential to cellular function and viability. DRP1 plays a pivotal role in maintaining this balance by mediating fission, a process critical to the removal of damaged mitochondria through mitophagy and the redistribution of mitochondrial content to meet localized cellular energy demands. Aberrant DRP1 activity, whether excessive or insufficient, has been reported to induce abnormal mitochondrial morphology, mitochondrial distribution, bioenergetic deficits, oxidative stress, apoptosis, and impaired autophagy-all of which may contribute to neurodegeneration and other diseases (Chan, 2020; Knott et al., 2008; Oettinghaus et al., 2012; Oliver and Reddy, 2019). Excessive mitochondrial fission with strategies used to reduce DRP1 function has been reported to be protective in experimental models of neurodegenerative diseases such as Alzheimer’s disease (AD) and PD (Oliver and Reddy, 2019). In preclinical models of PD, pharmacological inhibitors (such as P110 and mdivi-1) and genetic manipulations (loss of DRP1 function or overexpression of fusion proteins) attenuate mitochondrial abnormalities and improve neuronal function and survival (Bido et al., 2017; Cui et al., 2010; Fan et al., 2019; Filichia et al., 2016; Lutz et al., 2009; Rappold et al., 2014; Su and Qi, 2013). Together, such studies indicate DRP1 as a potential therapeutic target for PD. However, to date, the expression of this protein and other mitochondrial fission/fusion proteins have not been examined in the brains of Parkinson’s patients.

In the present study, we analyzed human post-mortem SNpc from 17 PD and 16 non-PD subjects for the expression of DRP1. Our data indicate that DRP1 is significantly increased in human Parkinson’s patients at both mRNA and protein levels. Furthermore, in these patients, immunoreactivity of DRP1 was higher in DA neurons, microglia and astrocytes, compared to those in non-PD individuals. Considering studies that have reported blocking DRP1 reduces neuroinflammation (Joshi et al., 2019; Park et al., 2013), our observations suggest reducing DRP1 levels may also attenuate neuroinflammation in PD, providing an additional neuroprotective mechanism. In addition to DRP1, we assessed the levels of other fission (*MIEF1*, *MIEF2*, *MFF*) and fusion (*OPA-1* and *MFN2*) genes. Consistent with the increased expression of *MIEF1* and *OPA-1* transcripts, we also detected an increase in their corresponding gene products MID51 and OPA-1. Because MID51 is a receptor for DRP1 (Pernas and Scorrano, 2016), their elevated levels would result in pro-fission. Perhaps OPA-1 is upregulated to compensate for this effect. Interestingly, we did not detect a similar change with another fusion gene *MFN2*, which has been reported to be reduced in patients with AD (Wang et al., 2009). Given the variance in age and sex between Parkinson’s patients and non-PD controls, samples were adjusted for age and sex. No sex differences were identified between any of the analyzed mitochondrial fission/fusion proteins. Age also did not significantly influence the levels of these proteins. However, this undetectable age-dependent effect may be due to the lack of the longitudinal aging aspect in our patient population, whose ages ranged only from 75 to 88 years old. A younger cohort would be required to evaluate if the alterations in mitochondrial fission/fusion proteins are age dependent. Taken together, both pro-fission (DRP1 and MID51) and pro-fusion (OPA-1) proteins were upregulated in Parkinson’s patients. One approach to determine whether such alterations would result in pro-fission or fusion phenotype is to quantify mitochondrial morphology. However, assessing mitochondrial morphology in human post-mortem samples is inherently challenging due to the rapid structural alterations that occur after death when organelles are assumed to cease functionality (Barksdale et al., 2010). Given that mitochondria are highly dynamic organelles, their morphology can be influenced by factors such as agonal state, post-mortem interval, and the tissue preservation methods, making it difficult to distinguish pathological changes to the mitochondria from artifacts (Barksdale et al., 2010; Harish et al., 2013).

To complement some of the inherent challenges such as the ones discussed above when using human brain samples and other concerns such as potential alterations to tissue integrity due to variability in postmortem interval, heterogeneity of the severity of the disease, sporadic *versus* genetic PD, and potential confounding factors such as medications taken and exposure to environmental toxins, we included a transgenic mouse model overexpressing human WT α-syn, which is involved in both familial and sporadic PD. Another advantage of using the *SNCA* mice is that these animals allowed us the feasibility to directly study the *in vivo* effects of α-syn on mitochondrial dynamics in an age- and sex-dependent manner. Our results show an upregulation of DRP1 in the 6-month-old *SNCA* mice when compared to other age groups and their age-matched WT littermate controls. Interestingly, we observed that aging by itself also increased the levels of this fission gene and protein at 6 months old, but to a lesser extent than when combined with α-syn. It is tempting to speculate that perhaps such alteration contributes to the age-dependent onset of PD. Mitochondrial fragmentation was observed in 6- and 12-month-old *SNCA* mice which was corroborated by increased mitochondrial colocalization of DRP1. These changes were observed in tandem with increases of pDRP1-s616 in TH-positive neurons of the SNpc in 6- and 12-month-old *SNCA* mice. We believe such an increase in post-translational modification of DRP1 contributed to mitochondrial fragmentation in 12-month-old *SNCA* mice, despite no changes in the total levels of DRP1 at this stage. The contribution of DRP1 to this phenotype was further corroborated whereby mitochondrial fragmentation was prevented in the DRP1-KO mice. We also observed dynamic changes in other pro-fission and fusion genes/proteins in the *SNCA* mice. Increased expression of pro-fission proteins, such as DRP1 and MID51, may lead to a compensatory change in the regulation of pro-fusion proteins such as MFN1, MFN2, and OPA-1 to maintain mitochondrial homeostasis and prevent pathological fragmentation. Alterations in both fission and fusion proteins have also been reported in AD patients and experimental models of AD (Kandimalla et al., 2022; Manczak et al., 2016; Wang et al., 2009).

No changes were detectable in any mitochondrial proteins between 3-month-old WT and *SNCA* mice. It is possible that at this early age α-syn pathology is still developing. Although total α-syn levels in these *SNCA* mice increase at 3-months-old, a minor increase in α-syn pS129 levels only began in 8- and 12-month-old mice (Choi et al., 2020; Polinski et al., 2022). It has been shown that in human Parkinson’s patients pathogenic aggregates of α-syn pS129 preferentially bind to mitochondria in contrast to their soluble counterparts (Ludtmann et al., 2018; Wang et al., 2019). Specifically, the α-syn N-terminus binds to lipids on the mitochondrial membrane, such as cardiolipin (Maldonado Vidaurri et al., 2018). Under pathophysiological conditions cardiolipin oxidizes and translocates from the inner to outer mitochondrial membrane (Chu et al., 2013). Certain post-translationally modified species of α-syn bind with high affinity to the TOMM20, resulting in preventing the interaction of this mitochondrial receptor with its co-receptor, TOMM22, and impaired mitochondrial protein import (Di-Maio et al., 2016). Thus, these interactions with α-syn can impact the import and export of proteins and ions at the mitochondria leading to mitochondrial stress and potentially fragmentation. Such previous studies combined with the data we show of increased pDRP1-s616 phosphorylation and subsequent mitochondrial fragmentation in PD models highlight the important role that α-syn may play in mediating DRP1-induced mitochondrial fragmentation in the older *SNCA* mice. Of note, pathogenic point mutations of α-syn has also been reported to induce mitochondrial fragmentation *via* a DRP1-independent mechanism (Guardia-Laguarta et al., 2014).

## 5. Conclusion

To our knowledge this is the first study that investigates mitochondrial fission and fusion factors in human Parkinson’s postmortem specimens, coupled with a transgenic mouse model overexpressing human α-syn. Our results highlight a dysregulation of mitochondrial fission and fusion proteins in both Parkinson’s patients and *SNCA* mice. However, we observed that not all alterations are consistent between human and mouse samples. MID51 and OPA-1 were increased in PD patients but not in the transgenic *SNCA* mice. Like other animal models of PD, we believe expressing α-syn alone cannot recapitulate all the complex factors that account for the neuropathology as seen in PD. Such factors may include comorbidities that we are unaware of, genetic makeup and environmental exposure during lifespan. Nonetheless, increased DRP1 levels were consistently observed in these patients and mutant mice. Relevant to this study, as discussed, blocking DRP1 has been reported to be neuroprotective against mitochondrial dysfunction, oxidative stress, impaired autophagy, aggregated α-syn, and synaptic dysfunction in PD models. The present study highlights DRP1 as an attractive target for PD and synucleinopathies.

## Supplementary Material

1

## Data Availability

Human post-mortem samples are available through NIH-NeuroBiobank. Transgenic mice expressing human *SNCA* under the *Thy-1* promoter [C57BL/6 N-Tg(Thy1-SNCA)15Mjff/J, Strain #017682] are commercially available from the Jackson Laboratory. Data are provided within the manuscript or supplementary information files

## Data availability

Data will be made available upon request.

## Appendix A. Supplementary data

Supplementary data to this article can be found online at https://doi.org/10.1016/j.nbd.2025.106976.
